# Omeg@Silica: Entrapment and Stabilization of Sustainably Sourced Fish Oil

**DOI:** 10.1002/open.202100038

**Published:** 2021-05-04

**Authors:** Rosaria Ciriminna, Claudia Lino, Mario Pagliaro

**Affiliations:** ^1^ Istituto per lo Studio dei Materiali Nanostrutturati, CNR via U. La Malfa 153 90146 Palermo Italy

**Keywords:** Omeg@Silica, natural carotenoids, periodic mesoporous silica, omega-3, anchovy, fish oil

## Abstract

Fish oil rich in long‐chain polyunsaturated fatty acids, vitamin D_3_ and carotenoid pigments have been sustainably extracted from anchovy fillet leftovers using biobased limonene. The oil is conveniently stabilized by adsorption on periodic mesoporous silicas. The simplicity of the process, the high load of fish oil, and the biocompatible nature of mesoporous silica support numerous forthcoming applications of this new class of “Omeg@Silica” materials.

## Graphical abstract

**Sustainable extraction**: Fish oil rich in long‐chain polyunsaturated fatty acids, vitamin D_3_ and carotenoid pigments have been sustainably extracted from anchovy fillet leftovers using biobased limonene. The oil is conveniently stabilized by adsorption on periodic mesoporous silicas. The simplicity of the process, the high load of fish oil, and the biocompatible nature of mesoporous silica support numerous forthcoming applications of this new class of “Omeg@Silica” materials.

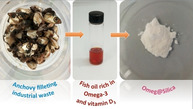

## 1 Introduction

Inuit populations living in Greenland in the early 1970s eating only whale, seal and fish meat presenting virtually no cardiovascular disease (and no diabetes mellitus) were found to have high levels of two omega‐3 long‐chain polyunsaturated fatty acids (PUFAs), docosahexaenoic acid (DHA) and eicosapentainoic acid (EPA), in their plasma and platelet lipids.^[1]^ Following this discovery and a large number of subsequent biochemical and clinical studies,^[2]^ fish oil has become the source of refined omega‐3 lipids used as active ingredients in a variety of dietary supplements. Multiple health benefits are ascribed to these lipids, starting from reduction of cardiovascular risk and tissue inflammation.^[3]^

To increase the dietary intake of omega‐3 lipids, whose recommended daily intake is not met by the population of most economically developed countries, marine oils rich in EPA and DHA are added also to “fortified” milk powders (including infant formulas),^[4]^ and to animal feed to produce omega‐3 enriched eggs.^[5]^ One of the main deficiencies in diets common to industrially developed countries, indeed, is the insufficient assumption of EPA and DHA.^[6]^ Increasing the intake of EPA+DHA essential fatty acids requires to either increase consumption of fish and seafood in the diet, or omega‐3 supplementation.

Widespread awareness of this diet deficiency^[6]^ has led to a global and growing demand of fish oil which, in its turn, is enhancing overfishing pressure on fish and krill stocks.^[7]^ Accordingly, shifting the production of fish oil, the main ingredient of omega‐3 dietary supplements, from fish to fish processing waste produced in huge yearly amount by the fish processing industry is an urgent challenge of global relevance.^[8]^ In this context, we recently reported a new circular approach to the production of high quality fish oil involving the solid‐liquid extraction of anchovy fillet leftovers using citrus‐derived *d*‐limonene as green bio solvent.^[9]^ The terpene, which is also an antibacterial and antioxidant,^[10]^ protects PUFAs in the fish oil from otherwise quick catalytic oxidation due to free radicals formed in the presence of air's oxygen,^[11]^ both during the fish oil extraction at room temperature and during solvent recovery by evaporation under reduced pressure at 90 °C.^[9]^

In general, fish oil in food supplement capsules first undergoes chemical refining and transformation into ethyl esters and then is added with natural or synthetic antioxidants prior to encapsulation in soft gelatin capsules.^[8]^ In other usages, microencapsulation techniques are used by industry to microencapsulate and protect fish oil from peroxidation.^[12]^

Now, we report the outcomes of encapsulating the newly extracted fish oil obtained from anchovy filleting industrial waste in periodic mesoporous silica particles. Both biocompatible MCM‐41 silica and organically modified mesoporous silica materials were used as adsorbent for this new fish oil rich not only in EPA and DHA^[9]^ but also in vitamin D_3_ ^[13]^ and natural carotenoids. To the best of our knowledge, the only fish oil microencapsulation process involving silica was reported by Australia's scholars in 2018.^[14]^ Spray drying a fish oil submicroemulsion stabilized by food‐grade hydrophilic silica particles, the team showed that microencapsulation in silica indeed protects the omega‐3 lipids from oxidation regardless of the harsh fabrication conditions (inlet temperature, 140 °C and high pressure) employed to spray dry the functionalized silica particles.

The outcomes of the material synthesis and structural investigation reported in this study suggest that this new class of materials, called herein “Omeg@Silica”, has the potential to expand the biological applications of omega‐3 lipids, from better fortified foods, through controlled release and synergistic effects with silica in biomedical uses.

## 2 Results and Discussion

**Figure 1:**
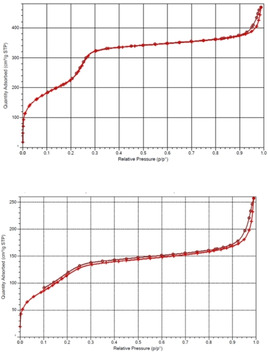
N_2_ adsorption‐desorption isotherms of MCM‐41 (top), and FMCM‐41 (bottom).

MCM‐41 silica was synthesized by the template‐assisted sol‐gel process using a published protocol,^[15]^ and subsequently functionalized with 3‐aminopropyl groups by post‐grafting.^[16]^ The inner porosity of MCM‐41 silica was able to encapsulate more oil (50 wt %) than aminopropyl‐silica FMCM‐41 (40 wt %). Figure 1 readily shows the reason. Both materials show type IV N_2_ adsorption‐desorption isotherms whose hysteresis loop of moderate steepness is associated with capillary condensation taking place in narrow, slit‐like mesopores.^[17]^ Data in Table 1 show evidence of the significant decrease of both specific surface area (SSA), specific pore volume (SPV) and average pore diameter of MCM‐41 upon grafting the internal pore walls with the aminopropyl groups.

**Table 1:** Textural properties of MCM‐41 and FMCM‐41.

Material	Specific surface area [m^2^/g]	Specific pore volume [cm^3^/g]	Average pore diameter [nm]
MCM‐41	825	0.6	2.9
FMCM‐41	440	0.3	2.5

Once loaded with 50 wt % sustainably sourced anchovy fish oil, the surface area of MCM‐41 measured by N_2_‐adsorption decreases to 16 m^2^/g (entry 1 in Table 2). Increasing the load of fish oil adsorbed by the aminopropyl‐functionalized silica from 10 to 40 wt %, the SSA and the SPV respectively go from 166 m^2^/g to 16 m^2^/g and from 0.1 cm^3^/g to 0.02 cm^3^/g (entries 2 and 5 in Table 2), showing that the adsorption of the fish oil takes place via entrapment of the triglyceride molecules in the inner porosity of the silicas.

**Table 2:** Textural properties of Omeg@Silicas comprised of MCM‐41 and FMCM‐41 loaded with different amount of sustainably extracted fish oil.

Entry	Material	Specific surface area [m^2^/g]	Specific pore volume [cm^3^/g]	Average pore diameter [nm]
1	MCM‐41_50 %	16	0.02	6.1
2	FMCM‐41_10 %	166	0.1	3.3
3	FMCM‐41_20 %	49	0.08	6
4	FMCM‐41_30 %	24	0.06	8.5
5	FMCM‐41_40 %	16	0.02	5.3

**Figure 2:**
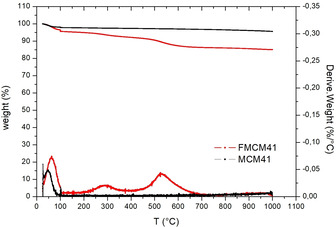
TGA and DTG curves of MCM‐41 (black) and FMCM‐41 (red).

The amount of aminopropyl‐trimethoxysilane grafted on the MCM‐41 silica surface was derived from the thermogravimetric analysis (TGA) of both MCM‐41 and FMCM‐41 displayed in Figure 2 along with the derivative thermogravimetry (DTG) curve.

Three weight losses were observed in the TGA curve of FMCM‐41. The first, close to 100 °C (−4.4 %), is due to evaporation of water adsorbed at the surface of hydrophilic silica. The second, at about 300 °C (−4.3 %), originates from the desorption of physically adsorbed APTMS.^[18]^ The third, above 430 °C (−6.2 %), is due to thermal decomposition of the −(CH_2_)_3_NH_2_ groups chemically bound to the silica structure, with the C−Si bond starting to break up at 450–510 °C.^[18]^

Following Wang and co‐workers,^[19]^ the mole number of APTMS grafted on the silica surface was calculated from the weight loss ΔW (%) at 430 °C expressed as *n*_A_ (mol)=m_430_/M_NH_ where m_430_ (g) is the weight loss of the sample at 430 °C (0.9513 mg), and M_NH_ (g/mol) is the molecular weight of the −(CH_2_)_3_NH_2_ group (58 g/mol). The APTMS content (mmol/g_silica_) is given by *n*_A_ divided by the silica mass (*m*_SiO2_) determined by subtracting the mass brought by grafted APTMS from the residual weight at 300 °C (*m*_300_). In the present case, the calculated amount of aminopropyl groups is 1.07 mmol/g, namely 76 % of the theoretical value (1.4 mmol/g), pointing to the fact that successful MCM‐41 functionalization via post‐grafting method.^[16]^

**Figure 3:**
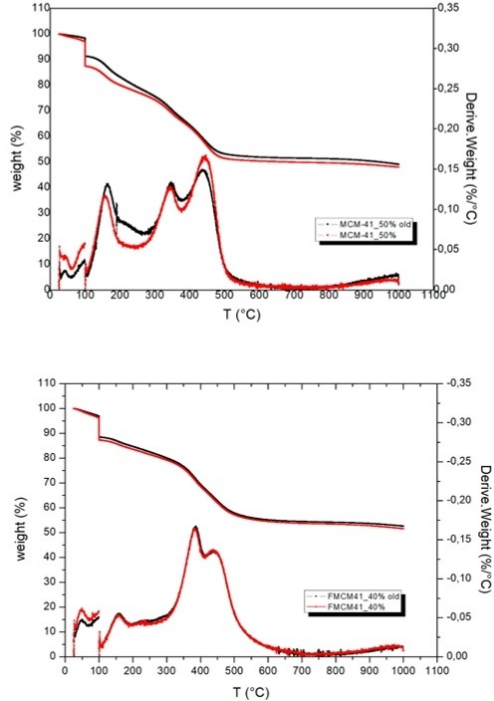
TGA and DTG curves of Omeg@Silicas MCM‐41_50 % (top), and FMCM‐41_40 % (bottom).

The TGA was used to assess also the thermal stability of the mesoporous silicas loaded with 50 wt % and 40 wt % anchovy fish oil, by carrying out the TGA measurements one month after storing the loaded materials at room temperature under conventional atmosphere. The overlapping curves in Figure 3 clearly show that both MCM‐41 loaded with 50 wt % oil and its aminopropyl‐functionalized counterpart doped with 40 wt % fish oil show excellent thermal stability over time.

**Figure 4:**
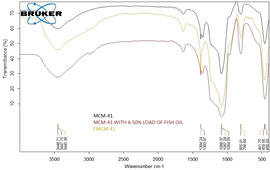
FT‐IR spectra of MCM‐41 (black), Omeg@Silica MCM‐41_50 % (red) and FMCM‐41 (gold).

Evidence of successful encapsulation of fish oil was obtained also from comparing the FTIR spectra of MCM‐41 and of the same material functionalized with 50 wt % anchovy fish oil. The spectrum of MCM‐41 (black curve in Figure 4) is typical of sol‐gel silica with the main infrared bands *ν*OH at 3200–3300 cm^−1^, *ν*_as_ Si−O−Si at 1080 cm^−1^, *ν*Si−OH at 950 cm^−1^ and *ν*_s_ Si−O−Si at 800 cm^−1^, and *ρ* Si−O−Si at 460 cm^−1^.^[20]^

The spectrum of MCM‐41 loaded with 50 wt % fish oil (red curve in Figure 4) clearly shows peaks in the fingerprint region (800–1500 cm^−1^), especially in the 1050–950 cm^−1^ region, which has been ascribed to =C−H out‐of‐plane bending.^[21]^ Remarkably, the C=O carbonyl band at 1720–1746 cm^−1^ normally significant in omega‐3 lipids, is absent in the latter spectrum which likely indicates coordination of the carbonyl's oxygen with the numerous Si−OH groups at the surface of the sol‐gel cages.

**Figure 5:**
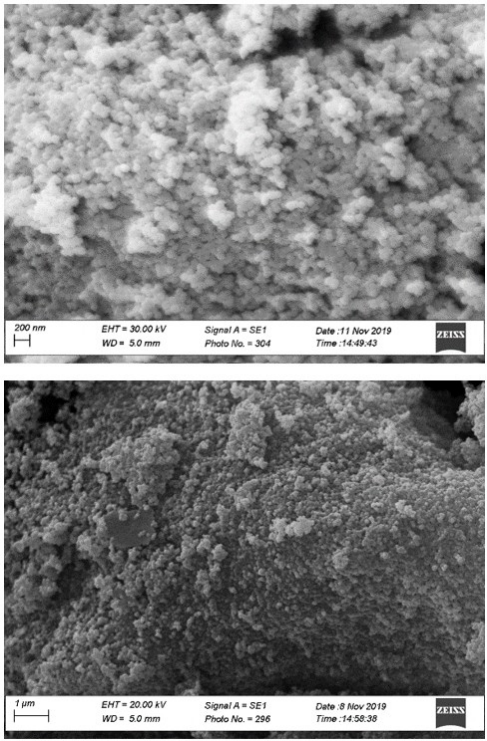
SEM microphotographs of MCM‐41 silica (top), and Omeg@Silica MCM‐41_50 % (bottom).

The SEM microphotographs (Figure 5) show that filling the inner porosity of MCM‐41 with 50 wt % fish oil changes the outer surface morphology of the mostly spherical SiO_2_ submicron particles into a smoother, more compact agglomerate of amorphous particles.

Finally, the zeta potential of MCM‐41 and FMCM‐41 Omeg@Silicas doped, respectively, with 50 wt % and with 40 wt % fish oil indicate (entries 2 and 7 in Table 3) pronounced stability of the aqueous suspensions of the materials. Showing evidence of the silica nature of the suspended particles,^[22]^ all suspensions have negative zeta‐potential. The 40.4 mV absolute value of zeta‐potential of the aminopropyl‐silica doped with 40 wt % anchovy fish oil (entry 7) points to its pronounced stability. On the other hand, the absolute values for suspensions of the same organically modified silica empty (entry 3) or doped with 10, 20 and 30 wt % fish oil (entries 4,5, and 6) indicate lack of stability towards aggregation.

**Table 3:** Zeta potential, Z‐Ave and PDI of MCM‐41 and FMCM‐41 and Omeg@Silicas loaded with different amounts of anchovy fish oil.

Entry	Material [1 mg/mL]	Zeta potential [mV]	Z‐Ave [nm]	PDI
1	MCM‐41	−33,4	217	0.3
2	MCM‐41_50 %	−37.6	269	0.3

	Material (0.5 mg/mL)			
3	FMCM‐41	−14.9	297	0.4
4	FMCM‐41_10 %	−8.67	1915	0.4
5	FMCM‐41_20 %	−23.7	1777	0.4
6	FMCM‐41_30 %	−29.4	1100	0.5
7	FMCM‐41_40 %	−40.4	522	0.5

We briefly remind that particles in suspension having a large positive (>30 mV) or negative (<‐30 mV) value of the zeta potential repel each other: aggregation is prevented and the dispersion remains electrostatically stable. In brief, functionalization with fish oil of both pure silica or organically modified silica is beneficial for the electrostatic stability of microparticles suspensions in water provided that high amounts of fish oil are loaded within the silica inner porosity.

**Figure 6:**
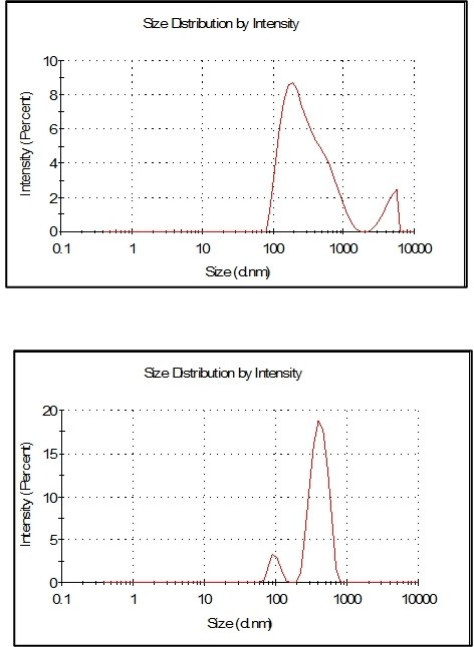
Particle size distribution of Omeg@Silica MCM‐41_50 % (top), and Omeg@Silica FMCM‐41_40 % (bottom).

The particle size distributions plotted in Figure 6 from dynamic light scattering (DLS) measurements indicate that the population of particles is represented by well dispersed submicron particles with size distributed around 269 nm for the MCM‐41_50 % Omeg@Silica, and around 522 nm for the aminopropyl‐modified FMCM‐41_40 % Omeg@Silica.

The materials respectively have a polydispersity index (PDI) lower (0.3 in the case of aminopropyl‐modified Omeg@Silica) or equal to 0.5 (in the case of unmodified Omeg@Silica), which is an important characteristic in drug delivery applications.^[23]^ The Z‐average size (measure of the average size of the particle size distribution resulting by DLS) indicates a moderate increase from 217 to 269 nm for MCM‐41 silica going from empty to filled with 50 wt % fish oil, and almost doubling in size (from 297 to 522 nm) for FMCM‐41 aminopropylsilica particles going from empty to particles Omeg@Silica with the highest 40 wt % load.

## 3 Conclusions

Expanding the uses of valued fish oil via microencapsulation and stabilization is of significant practical relevance. In this study, we show how microencapsulation of sustainably sourced fish oil is readily and easily achieved by adsorption over periodic mesoporous silicas. Both mesoporous MCM‐41 silica and FMCM‐41 aminopropyl‐silica submicroparticles are excellent biocompatible supports for adsorption and delivery of whole fish oil rich in omega‐3, vitamin D_3_ and natural carotenoids (*AnchoisOil*) sustainably sourced from anchovy filleting industrial waste using biobased limonene only.^[9]^

The entrapment process is simple and takes place at room temperature and pressure with no need for microencapsulation devices. It is enough to add the newly extracted fish oil to the as‐synthesized material kept under agitation at room temperature to rapidly obtain a silicate powder incorporating a high load of marine oil.

**Figure 7:**
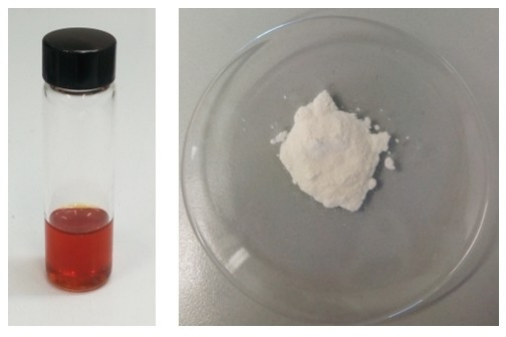
Oil extracted from anchovy filleting waste (left), Omeg@Silica comprised of MCM‐41 loaded with 50 % w/w fish oil (right).

Quick and effective oil entrapment of the oil is due to the huge specific surface area and pore volume of both silicas whose ordered hexagonal pores act as “chemical sponges”, adsorbing and concentrating the lipid, vitamin D_3_ and carotenoid molecules within the inner mesoporosity. The outcome is a white and free flowing powdered material series dubbed herein “Omeg@Silica” (Figure 7).

The large demand of omega‐3 lipids, whose main ingredient used by the nutraceutical and dietary supplement industries is fish oil, is accelerating depletion of several marine species,^[7]^ including anchovy, one of the most fished species across the world. Sourcing fish oil from fish processing waste rather than from fish is an urgent and global economic and environmental challenge of global relevance.^[8]^ In a series of papers opened by the discovery of the extraction process using biobased limonene,^[9]^ we have jointly shown how fish oil sourced from anchovy or shrimp^[24]^ industrial waste has high nutraceutical value,^[13]^ and that the extraction method is technically and economically viable.^[25]^

In general, periodic mesoporous silicas are biocompatible and suitable for the targeted release of bioactive molecules in the gastrointestinal tract,^[26]^ even though mesoporous silica comprising the MCM‐41 structure is rapidly degraded under *in vitro* digestion conditions, while organically functionalized silica with 3‐amino‐propyl groups is generally more stable.^[27]^

Due both to larger interfacial surface area of silica‐entrapped fish oil triglyceride molecules and to the interfacial effect of the silica cages guiding access of the external reactants (including enzymes) to the entrapped species,^[28]^ once entrapped in mesoporous silicas omega‐3 long‐chain polyunsaturated fatty acids in triglyceride form are even more bio accessible to lipase (40 % faster lipolysis kinetics ascribed to the lipase molecules adsorbed to the silica surface in their active, open‐lid conformation)^[29]^ in comparison to crude fish oil.

All this suggests that Omeg@Silica materials of facile preparation and handling are suitable for multiple forthcoming practical applications, ranging from fortified foods to controlled release and delivery of omega‐3 lipids protected from oxidation and biological degradation hopefully leading to synergistic effects with the encapsulant mesoporous silica in biomedical uses.

## Experimental

### Fish oil extraction

Fish oil was extracted from anchovy filleting industrial waste kindly donated by an industry based in Sicily (Agostino Recca Conserve Alimentari, Sciacca) using *d*‐limonene as green bio solvent according to the published solid‐liquid extraction and solvent recovery processes.^[9]^

The fatty acid composition of anchovy discard fish oil was assessed following the standard method involving trans‐esterification of the oil triglycerides and CG‐MS analysis of the respective fatty acid methyl esters using a ThermoScientific Trace 1310/ISQ LT single quadrupole GC/MS spectrometer. The marine oil extracted was found to be rich in omega‐3 eicosapentaenoic acid (EPA) and docosahexaenoic acid (DHA, Table 4).

**Table 4:** EPA and DHA in fish oil extracted from anchovy waste.

Polyunsaturated Fatty Acid (in lipid numbers)	Abundance
EPA (20 : 5, *n*‐3)	5.4 %
DHA (22 : 6, *n*‐3)	12.4 %

### Synthesis and Characterization of the Materials

In detail, 1 g of hexadecyltrimethylammonium bromide (CTAB≥99 % pure, Aldrich) and 280 mg of sodium hydroxide (Analyticals, Carlo Erba) were dissolved in 480 mL of deionized water. An aliquot (5.4 mL) of tetraethylorthosilicate (TEOS≥99 % pure, Aldrich) was added dropwise to the solution. The resulting mixture was kept under continuous mechanical stirring (400 rpm) at 80 °C for 2 h. The solid precipitate was recovered by filtration, washed with abundant deionized water and methanol (99.8 % pure, Aldrich) and dried at 60 °C for 48 h. The residual surfactant entrapped in the silicate was removed via calcination at 550 °C for 6 h. In detail, an aliquot (600 mg) of the as‐synthesized MCM‐41 was refluxed in 15 mL toluene (≥99.5 % pure, Fluka Chemika) added with 152 μL of 3‐amino‐propyltrimethoxysilane (APTMS, 0,42 mmol, 97 % pure, Alfa Aesar). The solution was kept under stirring for 6 h after which it was decanted. The solid product thereby obtained was washed three times with 15 mL of isopropanol (99 % pure, J.K. Baker, Fisher Scientific), and dried at 40 °C for 3 h.

Loading of the MCM‐41 and of the MCM‐41 organically functionalized with 3‐amino‐propyl groups (FMCM‐41) was carried out by direct adsorption of the oil by adding dropwise the oil to the material kept in a glass flask under agitation. For example, addition of a first 60 μL aliquot of fish oil to FMCM‐41 (100 mg), was followed by additions of 20 μL aliquots. After 8 min, addition of oil was terminated, and the material left under agitation for 24 h. In detail, MCM41 was loaded up to 50 % w/w fish oil (MCM‐41_50 %) (Figure 7), while FMCM41 could be loaded up to 40 % w/w (FMCM‐41_40 %).

The materials were characterized by FT‐IR spectroscopy (Bruker, ALPHA model), scanning electron microscopy (SEM) and thermogravimetric analysis (Mettler Toledo TGA/DSC). Surface area and porosity were determined from cryogenic nitrogen adsorption‐desorption analysis using a Micromeritics ASAP 2020 Plus 1.03 porosimeter. The SEM analyses were conducted with a SEM Zeiss EVO MA10 microscope. Particle size, polydispersity index (PDI) and zeta‐potential were measured with a Zetasizer Nano ZS (Malvern Instrument, United Kingdom) using Dynamic Light Scattering (DLS) after dispersing material samples in distilled water.

## Author information

The authors declare no competing financial interest.

## Conflict of interest

The authors declare no conflict of interest.
